# Ultrasound-Guided Versus Freehand Fine Needle Aspiration: A Comparative Study in Diagnosing Non-Salivary, Non-Thyroidal Head, and Neck Masses

**DOI:** 10.7759/cureus.82830

**Published:** 2025-04-23

**Authors:** Senthil K Rajamanickam, Eduardo Prades Morera, Anurag Agarwal, Shruti Senthilkumar, Nora Elsaid, Hisham Zeitoun

**Affiliations:** 1 Otolaryngology - Head and Neck Surgery, Betsi Cadwaladr University Health Board, Bangor, GBR; 2 General Surgery, Betsi Cadwaladr University Health Board, Bangor, GBR; 3 Otolaryngology, Birmingham Medical School, Birmingham, GBR; 4 Otolaryngology, Betsi Cadwaladr University Health Board, Bangor, GBR

**Keywords:** fine needle aspiration cytology (fnac), freehand technique, head and neck mass, non-salivary non-thyroidal mass, ultrasound (us)

## Abstract

Background

Fine needle aspiration cytology (FNAC) is widely used to evaluate head and neck masses, with both ultrasound (US)-guided and freehand techniques being employed. While US-guided FNAC is thought to enhance accuracy, freehand FNAC remains commonly used due to its practicality and speed. This study compares the diagnostic performance of US-guided and freehand FNAC, evaluating their sensitivity, specificity, accuracy, inconclusive rates, and time to histological confirmation.

Aim of the study

The aim of this study is to compare the inconclusive rates and time to histological confirmation of US-guided versus freehand FNAC in assessing non-salivary, non-thyroidal head, and neck masses.

Methods

A retrospective analysis of 439 FNAC reports (2012-2016) was conducted. Diagnostic metrics, including sensitivity, specificity, positive predictive value (PPV), negative predictive value (NPV), accuracy, inconclusive rates, and median time to histological confirmation, were analyzed.

Results

Among 439 FNAC procedures, 294 were US-guided and 145 were freehand. US-guided FNAC had a lower inconclusive rate (11.6% vs. 17.9%; p = 0.068) and higher specificity (61% vs. 38%), while sensitivity was comparable (91% vs. 95%). Accuracy was 81.6% for US-guided FNAC and 86.3% for freehand FNAC. Freehand FNAC had a shorter median reporting time (29 vs. 42 days).

Conclusions

Both techniques demonstrated similar diagnostic accuracy. While US-guided FNAC reduced inconclusive rates and improved specificity, freehand FNAC provided faster results, aiding quicker clinical decision-making. These findings suggest that both techniques have distinct benefits, and their use should be tailored to individual patient needs and clinical settings.

## Introduction

The evaluation of head and neck masses is crucial in otolaryngology and oncology, as early and accurate diagnosis optimizes patient outcomes. While most patients present with a palpable neck lump, approximately 15% are incidentally detected through imaging [1]. Fine needle aspiration cytology (FNAC) is the preferred initial diagnostic tool due to its minimally invasive nature, high accuracy, and safety. It distinguishes benign from malignant lesions, guiding treatment decisions. However, inconclusive results can delay management, necessitating further testing [2].

FNAC can be performed using freehand or ultrasound (US)-guided techniques. Freehand FNAC is commonly used in outpatient settings for palpable masses due to its convenience. However, concerns exist regarding its diagnostic yield, particularly in deep or non-palpable lesions, where sampling errors may occur. US-guided FNAC enhances precision, improves sample adequacy, and aids in lesion characterization, particularly in lymph node assessment [3,4].

Over the years, head and neck surgeons have increasingly performed US-guided FNAC without radiologists, improving efficiency and reducing delays [5]. At our institution, it is routinely used for parotid lesions and increasingly for other masses. This study aims to compare the conclusiveness and diagnostic turnaround time of freehand versus US-guided FNAC for non-salivary, non-thyroidal head, and neck masses. Additionally, we explore a sequential approach, using freehand FNAC initially and reserving US guidance for inconclusive cases, to expedite diagnosis and treatment. The findings from this study may refine clinical pathways and optimize diagnostic strategies in head and neck oncology.

## Materials and methods

A retrospective observational analysis was conducted at a large district general hospital, evaluating the diagnostic accuracy of freehand FNAC versus US-guided FNAC in the assessment of non-salivary, non-thyroidal head, and neck masses.

Patient data were retrospectively retrieved from the hospital’s cytology reporting system, including all consecutive FNAC reports from lymph node mass in the neck, between January 2012 and December 2016, across three different hospitals within the same district and health board. The selected timeframe (2012-2016) coincides with the transition from freehand FNAC to US-guided FNAC. This transitional phase offered a unique opportunity to evaluate and compare both techniques within the same healthcare system. FNAC procedures were performed in patients with clinically or radiologically suspected lymph node masses. Only FNACs from non-salivary, non-thyroidal head, and neck lesions were included. Patient demographics, including age, gender, cytology reports, pathology reports, and US findings, were recorded. Age was calculated from the date of birth to the date of the cytology request.

Freehand FNAC was performed by consultant head and neck surgeons on palpable lymph node masses, while US-guided FNAC was conducted by consultant radiologists. The choice of technique was based on the clinical palpability of the lesion. FNAC procedures were carried out using a 23-gauge needle attached to a 10-mL syringe, with or without aspiration. A minimum of two passes were performed in each case. No cytologist or pathology technician was present at the time of the FNAC to assess sample adequacy. The obtained aspirates were spread onto glass slides, air-dried, and stained with May-Grünwald-Giemsa (MGG). Residual material was processed as a cytoblock. FNAC results were classified as per the WHO Reporting System for Lymph Node Cytopathology [6]. The final diagnosis of malignancy was based on histopathological analysis where available. In cases without histological confirmation, TNM staging criteria for nodal metastasis were used as the reference standard.

**Figure 1:**
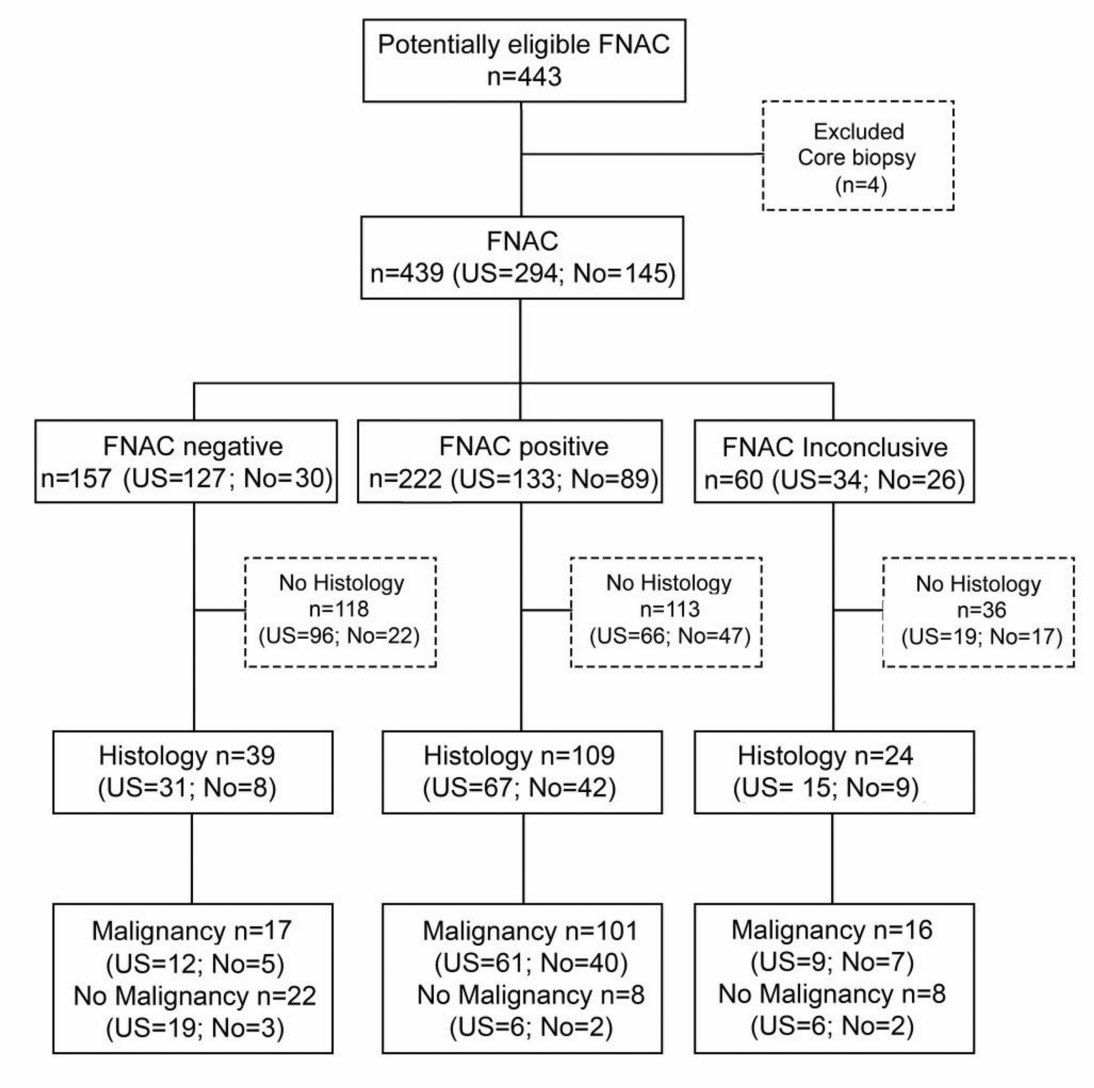
STARD diagram reporting flow of participants through the study. FNAC, fine needle aspiration cytology; STARD, Standards for Reporting of Diagnostic Accuracy Studies

The study’s primary outcome was the proportion of inconclusive FNAC results, including inadequate, non-diagnostic, or indeterminate findings, and the need for secondary FNAC. These outcomes were compared between freehand and US-guided FNAC groups. Secondary outcomes included sensitivity, specificity, accuracy, positive predictive value (PPV), and negative predictive value (NPV), which were assessed only in patients with confirmed histopathological results. The diagnosis of malignancy was used as the index test, with histopathological findings serving as the reference standard. Indeterminate FNAC cases were grouped with inadequate results under the inconclusive category for statistical analysis. Recommendations from the Standards for Reporting of Diagnostic Accuracy Studies (STARD) have been followed, as depicted in Figure 1 [7].

Indeterminate FNAC included cases where cytological features are suspicious but not definitive for malignancy or where reactive and neoplastic processes cannot be distinguished. Inadequate FNAC included cases that failed to meet the minimum cellularity or quality criteria required for a meaningful cytological assessment. Inconclusive rate is the proportion of FNAC procedures that do not provide a definitive diagnosis, requiring additional diagnostic steps. This includes both inadequate and indeterminate results. Core biopsy is a minimally invasive diagnostic procedure in which a hollow needle is used to extract a cylindrical sample of tissue from a suspected lesion for histopathological examination.

The differences in proportions of inconclusive FNAC results between US-guided and freehand groups were compared using a Z-test. A 95% confidence interval (CI) for the difference was calculated, with significance set at p < 0.05. Sample size was determined for two-tailed analysis with an expected difference of 10% between groups, an alpha level of 0.05, and 80% power. The interquartile range (IQR) is a measure of statistical dispersion that represents the range within which the middle 50% of a dataset lies. The target sample size was 438 FNAC cases (219 per group). Post-hoc analysis was performed to compare the need for secondary FNAC between the two groups, with Bonferroni correction applied for multiple comparisons. All statistical analyses were performed using STATA 15.0 statistical software (StataCorp LLC, College Station, TX, USA) and Microsoft Excel (Microsoft Corp., Redmond, WA, USA). No blinding was used in data or statistical assessment.

According to the NHS tool from the Medical Research Council, NHS REC review for sites in Wales was not required. No ethical approval was required for retrospective data from the care team under NHS policy.

## Results

A total of 443 reports were available, of which four were core biopsies. The study included 439 FNAC results, with 294 being US-guided and 145 freehand procedures. Among US-guided FNAC, 24.8% were positive for malignancy, while 31.7% of freehand FNAC were malignant. The inconclusive rate was 11.6% (9.2% inadequate, 2.4% indeterminate) for US-guided FNAC and 17.9% (13.8% inadequate, 4.1% indeterminate) for freehand FNAC.

Of the negative FNAC results, 24.8% had confirmed histology available (24.4% US-guided, 26.7% freehand), while 49.5% of positive FNAC results had confirmed histology (50% US-guided, 48.3% freehand). For inconclusive FNAC results, 40% had confirmed histology available (44.1% US-guided, 34.6% freehand).

Of the 439 FNAC cases, 205 were female (46.7%, mean age 60.8 ± 18.4 years), with 145 US-guided FNAC (49.3%, mean age 59.8 ± 17.9 years) and 60 freehand FNAC (41.4%, mean age 64.4 ± 19.5 years). The remaining 234 were male (53.3%, mean age 63.4 ± 17.1 years), with 149 US-guided FNAC (50.7%, mean age 62.6 ± 17.8 years) and 85 freehand FNAC (58.6%, mean age 64.0 ± 15.5 years). The age range of patients was 13 to 98 years.

Of the 118 benign FNAC diagnoses without confirmed histology, reactive lymph nodes were the most common (70.3%), followed by non-specific lymph node content (14.4%). For the 112 malignant FNAC diagnoses without confirmed histology, the most common diagnoses were squamous cell carcinoma ([SCC] 49.1%) and non-Hodgkin’s lymphoma (14.3%), as depicted in Table 1.

**Table 1:** Cytologic diagnosis of lymph nodes without histopathologic diagnosis. Note: % indicates the value in percentages, *n* indicates frequency

Benign (*n*=118)	n	%
Reactive lymph node	83	70.3
Non-specific lymph node content	17	14.4
Granulomatous lymphadenitis	10	8.5
Cyst	5	7.1
Warthin’s tumor	1	0.8
Abscess	1	0.8
Mucocele	1	0.8
Malignant (*n*=112)	n	%
Squamous cell carcinoma	55	49.1
Non-Hodgkins lymphoma	16	14.3
Poorly differentiated carcinoma	14	12.5
Adenocarcinoma	10	8.9
Melanoma	4	3.6
Malignancy unspecific for diagnosis	4	3.6
Small cell carcinoma	2	1.9
Lymphoma unspecific	2	1.9
Small cell vs non-Hodgkin’s lymphoma	1	0.9
Hodgkin’s lymphoma	1	0.9
Merkel cell carcinoma	1	0.9

A total of 39 reports had confirmed histology after a negative FNAC for malignancy. The most common cytologic diagnosis was reactive lymph node, accounting for 64.1%, followed by granulomatous lymphadenitis at 17.9%. After a positive FNAC for malignancy, 109 reports had confirmed histology. The most common cytologic diagnosis was SCC (48.6%), followed by non-Hodgkin’s lymphoma (21.1%). Additionally, 24 reports had confirmed histology following an inconclusive FNAC, with the most common malignant diagnosis being non-Hodgkin’s lymphoma (29.2%), followed by SCC (20.8%). Duplicate FNACs with the same histology were excluded from the final histopathologic diagnosis of parotid gland tumors (Table 2).

**Table 2:** Histopathologic Diagnosis of Lymph Nodes. Note: % indicates the value in percentages, *n* indicates frequency

Benign (*n*=32)	*n*	%
Reactive lymph node	18	56.3
Non-specific lymph node content	5	15.6
Granulomatous lymphadenitis	4	12.5
Angioleiomyoma	1	3.1
Schwannoma	1	3.1
Arteriovenous hemangioma	1	3.1
Warthin’s tumor	1	3.1
Lipoma	1	3.1
Malignant (*n*=107)	*n*	%
Squamous cell carcinoma	52	48.6
Non-Hodgkin’s lymphoma	31	29.0
Hodgkin’s lymphoma	8	7.5
Melanoma	5	4.7
Adenocarcinoma	3	2.8
Thyroid carcinoma	3	2.8
Mucoepidermoid carcinoma	1	0.9
Atypical fibrohistiocytoma	1	0.9
Small cell neuroendocrine carcinoma	1	0.9
Undifferentiated carcinoma	1	0.9
Basal cell carcinoma	1	0.9

A total of 139 (38.8%) definitive histology results were obtained from 358 patients. Of these, 92 (66.2%) patients underwent excision of one or more lymph nodes, 44 (31.7%) had one or more biopsies of the primary lesion, 5 (5.4%) had histology from a lesion suspected to be a lymph node during FNAC, 1 (1.1%) had a core biopsy, and 1 (1.1%) had histology of a distant metastasis.

The overall median time between FNAC and the availability of histology was 38.5 days, with 42 days for US-guided FNAC and 29 days for freehand FNAC. The time to obtain definitive histology varied by FNAC result. For negative FNAC, the median was 58 days, with an interquartile range (IQ25-IQ75) of 40 to 112 days. For inconclusive FNAC, the median was 42.5 days (IQ25-IQ75: 24-96). For positive FNAC, the median was 32.5 days (IQ25-IQ75: 21-53).

The main analysis revealed that the proportion of inconclusive FNAC (inadequate sampling or indeterminate results) was significantly smaller for US-guided FNAC (11.6%) compared to freehand FNAC in the clinic (17.9%). The difference in proportion (6.4%, 95% CI: 0 to 14.1) was not statistically significant (p=0.068).

The diagnostic accuracy measures for both US-guided and freehand FNAC showed similar results. The specificity of US-guided FNAC was higher (61%) compared to freehand FNAC (38%), while the sensitivity was similar for both methods (91% for US-guided and 95% for freehand FNAC) (Table 3).

**Table 3:** Measures of diagnostic accuracy and inconclusive proportion among lymph node masses. All values are presented in % (95% confidence interval). ^1^Total FNAC=439 (US-guided FNAC=294; freehand FNAC=145) FNAC, fine needle aspiration cytology; US, ultrasound

	Total FNAC (n=149)	US-guided FNAC (n=98)	Freehand FNAC (n=51)
Sensitivity	92.7 (86.1-96.8)	91.0 (81.5-96.6)	95.4 (84.2-99.4)
Specificity	56.4 (39.6-72.2)	61.3 (42.3-78.2)	37.5 (8.52-75.5)
Positive predictive value	85.7 (80.7-89.6)	83.6 (76.4-88.9)	89.1 (82.7-93.8)
Negative predictive value	73.3 (57.2-85.0)	76.0 (58.4-87.7)	60.0 (22.9-88.4)
Accuracy	83.2 (76.2-88.8)	81.6 (72.5-88.7)	86.3 (73.7-94.3)
Positive likelihood ratio	2.13 (1.48-3.05)	2.35 (1.50-3.69)	1.53 (0.89-2.62)
Negative likelihood ratio	0.13 (0.06-0.27)	0.15 (0.06-0.33)	0.12 (0.02-0.63)
Inconclusive^1^	13.7 (10.8-17.2)	11.6 (8.39-15.7)	17.9 (12.5-25.0)
Secondary FNAC^1^	8.42 (6.17-11.4)	9.18 (6.39-13.0)	6.90 (3.79-12.2)

A post-hoc analysis was conducted to assess the difference in the proportion of inconclusive results across three subgroups: patients diagnosed with lymphoma, SCC, or reactive hyperplasia. The cytology report was used when no histology was available. Bonferroni correction was applied, with significance set at 0.017. The results showed no significant differences in the proportion of inconclusive reports for lymphoma (US 11.6% vs non-US 17.4%), with a difference of 5.8% (95% CI: -10.8 to 26.6; p=0.51). Similarly, no significant differences were found in the SCC subgroup (US 2.9% vs non-US 7.4%), with a difference of 4.5% (95% CI: -4.0 to 14.8; p=0.26). For the reactive hyperplasia subgroup, no significant differences were observed (US 9.9% vs 18.1%), with a difference of 8.2% (95% CI: 0 to 17.8; p=0.05). Results from secondary FNAC showed no significant difference between the US and non-US groups.

## Discussion

Patients with head and neck cancers in England and Wales experience prolonged delays from referral to treatment initiation. The National Institute for Health and Care Excellence (NICE) recommends a target of 62 days to prevent tumor progression and improve prognosis. However, between 2018 and 2020, only 61% of patients with head and neck cancer met this target [8], underscoring the need for pathway improvements. One-stop clinics, incorporating ENT evaluation, US-guided fine needle aspiration cytology (USFNAC), and same-day histopathology, are advocated to reduce diagnostic timelines but are difficult to implement due to resource constraints [9]. As a result, FNAC remains the primary diagnostic tool, with either US- or palpation-guided techniques (palpation-guided FNAC [PGFNAC]) being used [10].

At our center, USFNAC typically takes two weeks following ENT referral, with histopathology requiring an additional week. When biopsy is unavailable, radiological evidence of metastasis per TNM staging, confirmed by multidisciplinary team consensus, serves as the gold standard. This approach has limitations, as lymph node histology often indicates lymphoma, while radiological positivity typically suggests metastasis. These assumptions and potential biases will be addressed in the discussion.

FNAC has been well-established as a highly effective diagnostic tool for head and neck masses. A 2008 systematic review [11] and subsequent studies [12] report a sensitivity of 92.5%, specificity of 97.8%, and accuracy of 94.3% for FNAC in lymph node masses [13]. However, there is a lack of systematic reviews directly comparing USFNAC and PGFNAC for non-thyroid, non-salivary gland masses. Retrospective studies, including those by Robinson and Cozens, Wu, and Horvath and Kraft [14-16], have examined these techniques, but direct comparisons with our study are challenging due to differing inclusion criteria.

Horvath and Kraft reported USFNAC sensitivity of 96% and PGFNAC sensitivity of 88% for lymph node masses, while our study found 91% and 95%, respectively. Accuracy rates in our study were 82% for USFNAC and 86% for PGFNAC, slightly higher than those reported by Robinson and Cozens (79% and 58%). Specificity values were lower in our study (61% USFNAC, 38% PGFNAC) compared to those reported by Horvath and Kraft (nearly 1 for both) and Wu (86% USFNAC, 50% PGFNAC). Our USFNAC inconclusiveness rate (12%) was lower than PGFNAC (18%), aligning with findings from Horvath and Kraft (9% USFNAC, 13% PGFNAC), Wu (14% USFNAC, 17% PGFNAC), and Robinson and Cozens (15% USFNAC, 33% PGFNAC). However, these results are not specific to non-thyroid, non-salivary gland masses, warranting further targeted research.

A large systematic review has shown that the overall inadequacy rate for US-guided USFNAC is 9.3% [17]. In our study, inadequacy rates were 9% for USFNAC and 14% for PGFNAC, aligning closely with the literature values. Ganguly et al. also reported a 9% inadequacy rate for USFNAC [17], while the average inadequacy rate for PGFNAC in the literature is 19% [18]. Robinson and Cozens found a lower inadequacy rate of 3% for USFNAC and a higher inadequacy rate of 22% for PGFNAC [15], whereas Wu reported even lower rates of 1% and 7%, respectively [14]. Ahn et al. attributed a 6.3% inadequacy rate for USFNAC in lymph nodes to factors such as cystic change and calcification, along with operator experience [13].

Our study found indeterminate rates of 2% for USFNAC and 4% for PGFNAC, lower than previous reports. Wu recorded 13% and 10% [14], while Robinson and Cozens reported 12% for both techniques [15]. Horvath and Kraft did not report inadequacy or indeterminate rates [16]. Despite USFNAC generally having lower inadequacy and indeterminate rates, statistical significance remains uncertain. A small randomized controlled trial by Robitschek et al. compared USFNAC and PGFNAC adequacy in diagnosing lymph node masses [19].

Lower inadequacy and indeterminate rates suggest that USFNAC may be more reliable for diagnosing non-malignant masses, particularly in cases without urgent diagnostic pressure. Tandon et al. reported an overall FNAC inadequacy rate of 15.2% and a non-diagnostic rate of 14.4% [10], higher than our study's findings. Selection bias in meta-analyses may contribute to lower reported inadequacy rates. Factors influencing FNAC adequacy include operator expertise, needle size, and on-site cytopathologist availability [13,17,20,21]. One-stop clinics, recommended by NICE, may enhance diagnostic efficiency [9]. There is also some evidence to suggest that non-diagnosed USFNAC cases may have a higher rate of malignancy compared to those non-diagnosed via PGFNAC [18], highlighting the potential clinical implications of FNAC inadequacy in this setting. The risk of malignancy in inadequate lymph node FNACs is well-documented, with some studies indicating a notable correlation [22]. While core needle biopsy generally demonstrates higher diagnostic accuracy than FNAC, it carries increased risks, including bleeding and potential tumor cell seeding [23].

This study adhered to the STARD checklist for diagnostic accuracy, with reference to the QUADAS tool, but had certain limitations [7]. Retrospective design and multiple operators introduced variability and potential selection bias, as patients with suspected malignancy were more likely to undergo PGFNAC, while ambiguous cases may be referred for USFNAC. Additionally, only 38.8% of FNAC cases had definitive histology, introducing further bias [24]. Verification bias was a concern as malignancy cases were more likely to proceed to surgery. The shorter median time to histology with PGFNAC (13 days) and its higher malignancy rate (32% vs. 25%) underscore the importance of reducing diagnostic delays.

While our findings do not support PGFNAC as the preferred method for diagnosing non-thyroid, non-salivary gland lesions based on accuracy alone, its faster diagnostic turnaround is a significant advantage. Delays in diagnosing lymph node malignancies can negatively affect outcomes. The growing adoption of USFNAC by head and neck surgeons, alongside radiologists’ expertise, is vital for improving diagnostic efficiency. One-stop clinics represent the ideal standard, but cost-effectiveness remains a challenge [9]. Thus, optimizing diagnostic pathways to minimize delays remains essential. Expanding surgeon training in US techniques may enhance the adoption of US-guided FNAC within head and neck clinics, ultimately reducing diagnostic delays and improving patient management pathways. A prospective study is under consideration within our department to formally evaluate an optimized, context-sensitive diagnostic strategy for head and neck masses. This proposed approach, tentatively named the SIMRAN (Strategic Integration of Methods for Rapid Assessment in Neck masses) approach, seeks to combine the advantages of both US-guided and freehand FNAC techniques. By tailoring technique selection to clinical context, lesion characteristics, and resource availability, SIMRAN aims to maximize diagnostic efficiency, reduce inconclusive rates, and streamline time to definitive histological confirmation.

## Conclusions

Freehand FNAC remains a safe and effective procedure, offering diagnostic accuracy comparable to US-guided FNAC while mitigating delays in cytology reporting. However, US-guided FNAC significantly reduces inconclusive results and the need for repeat procedures, particularly in the absence of concurrent cytological assessment. The US-guided FNAC neck lump clinic, with immediate sample evaluation, remains the gold standard for diagnostic accuracy, yet its widespread implementation is limited due to resource constraints. Prospective studies, including the one proposed in our department, may further clarify and support the benefits of this optimized approach, especially in resource-limited settings.
